# Retrograde and Contralateral Metastasis of Clear Cell Renal Cell Carcinoma to the Spermatic Cord: A Case Report

**DOI:** 10.7759/cureus.90970

**Published:** 2025-08-25

**Authors:** Joy M Hoang, Munira Charania, Marcus L Quek, Maria M Picken

**Affiliations:** 1 Department of Pathology, Loyola University Medical Center, Maywood, USA; 2 Department of Radiology, Loyola University Medical Center, Maywood, USA; 3 Department of Urology, Loyola University Medical Center, Maywood, USA

**Keywords:** clear cell renal cell carcinoma, contralateral, metastasis, renal cell carcinoma (rcc), retrograde, spermatic cord

## Abstract

Clear cell renal cell carcinoma (ccRCC) is known to display synchronous and metachronous metastases to a diverse set of organs. Retrograde and contralateral metastases of ccRCC are rare, with few cases in the literature. The mechanism of spread in these cases is thought to be facilitated by either the direct extension of tumor into the renal and gonadal veins or by the vertebral venous plexus. Here, we present a rare case of contralateral and retrograde ccRCC metastasis with extensive venous involvement.

## Introduction

Clear cell renal cell carcinoma (ccRCC) is the most common histologic subtype of renal cell carcinoma, accounting for about 75-80% of renal cell carcinomas, and frequently displays synchronous and metachronous metastases to the lungs, lymph nodes, bone, liver, and adrenal gland [1,2]. Metastasis in ccRCC occurs via hematogenous spread, as described in the literature noting direct venous extension and circulating tumor cells [3,4]. However, retrograde metastasis, defined as metastasis occurring venously downstream to the primary tumor, is rare, and contralateral retrograde metastases are extremely rare. In the literature, only 13 cases of ccRCC with metastasis to the spermatic cord have been reported in the literature, of which only one was contralateral [5,6].

This retrograde venous spread is postulated to be facilitated by the backwards flow through the spermatic cord vein from the renal vein [7]. This theory is supported by the paucity of contralateral metastases, the dominance of left-sided spermatic cord metastases, and the observation of direct tumor extension into the spermatic cord vein. The vertebral venous plexus, a valveless venous network extending from the spine to the pelvis, is a known facilitatory route of overall tumor spread. We postulate that it may also serve as a route of retrograde dissemination in ccRCC, especially in patients with contralateral metastasis. 

In this paper, we present a case of right kidney ccRCC with retrograde, contralateral metastasis to the left spermatic cord. 

## Case presentation

**Figure 1:**
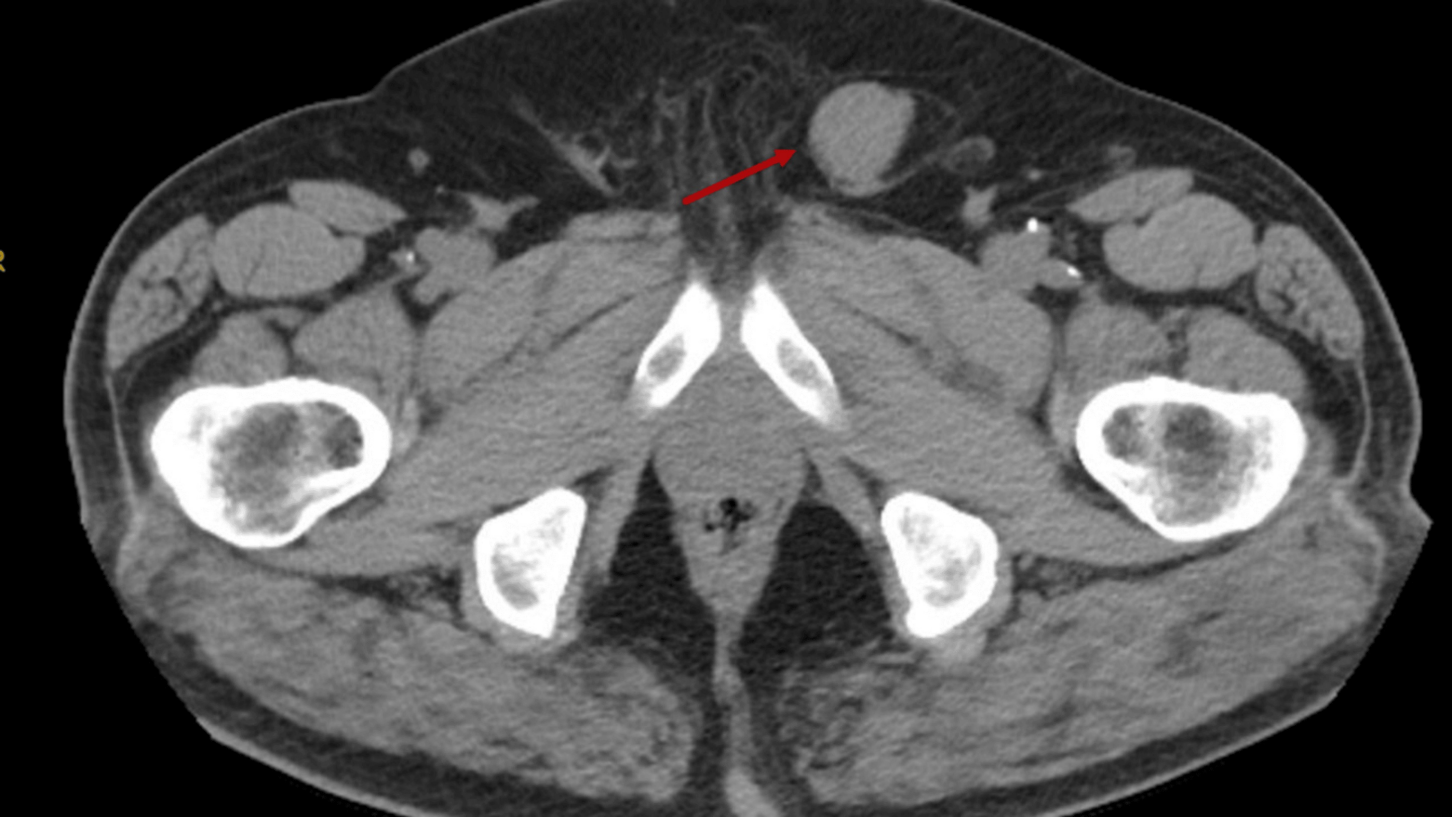
CT Abdomen and Pelvis A stable soft tissue density material is visualized within the left spermatic cord.

The patient is a 78-year-old man diagnosed three years prior in March 2022 with ccRCC, histologic grade three with invasion into the renal sinus with an overall stage of T3aN0Mx. He was treated with right-sided radical nephrectomy. He presented three years later with a growing, uncomfortable left inguinal mass; no constitutional symptoms were reported. Physical exam revealed a firm, mobile, non-transilluminating inguinal mass as well as bilaterally descended, symmetrical testes and left varicocele. This mass was previously noted 16 months prior on CT imaging and was characterized as a soft tissue density within the left spermatic cord (Figure 1). At the time, the mass was favored to be a varicocele. Repeat CT imaging in March 2025 showed a stable soft tissue density within the left spermatic cord. Follow-up scrotal ultrasound showed a solid, heterogeneous structure with vascularity measuring 7.8 x 2.7 x 2.0 cm. Based on the patient's clinical history and ultrasound findings, he underwent subsequent left radical orchiectomy with excision of the mass.

**Figure 2:**
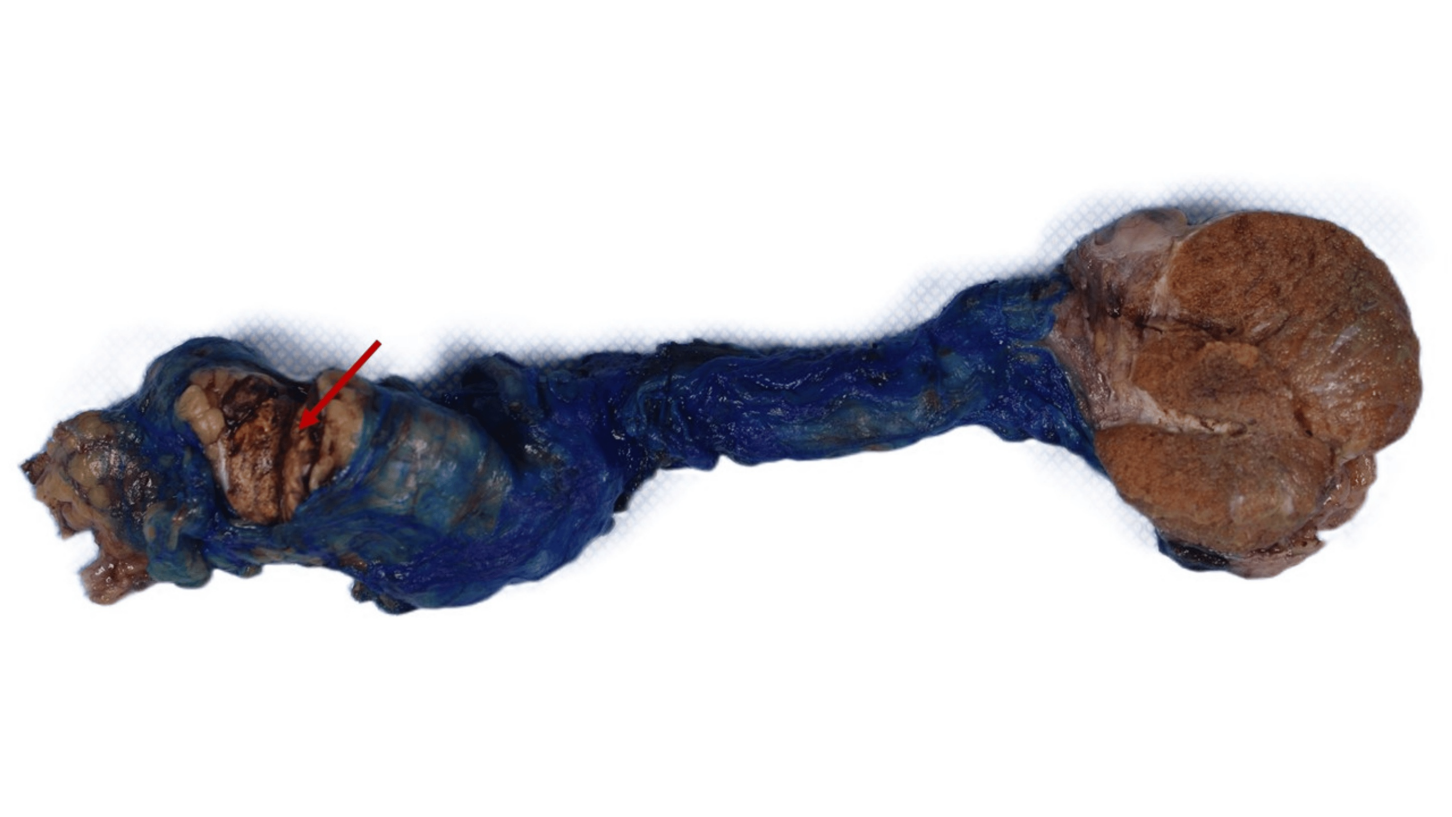
Intact Gross Specimen Gross examination upon opening the spermatic fascia shows a well-defined, golden-yellow mass with focal hemorrhage located within the spermatic cord. The testicle appears grossly normal.

**Figure 3:**
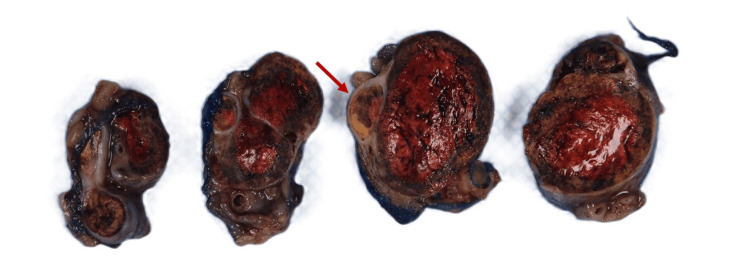
Sectioned Gross Specimen Gross examination upon sectioning the spermatic cord displays the yellow-tan mass contained within the spermatic cord and filling the associated venous structures.

**Figure 4:**
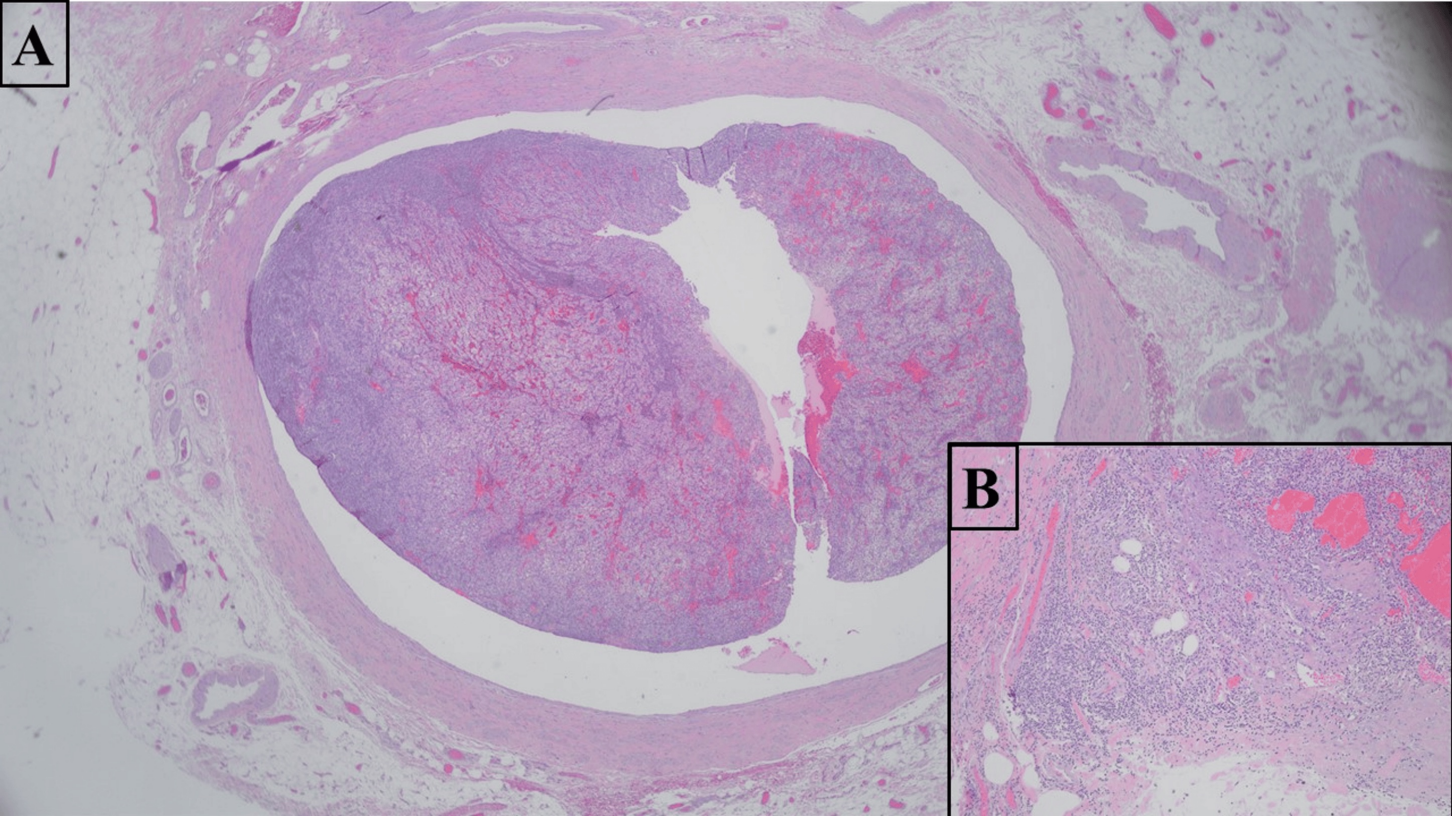
Microscopic View of the Tumor Using H&E Stain A. H&E, 20x. The tumor is identified within a large vein. B. H&E, 100x. Tumor with extravenous extension into surrounding soft tissue. Cells with abundant, clear cytoplasm and associated chicken-wire vasculature are seen.

**Figure 5:**
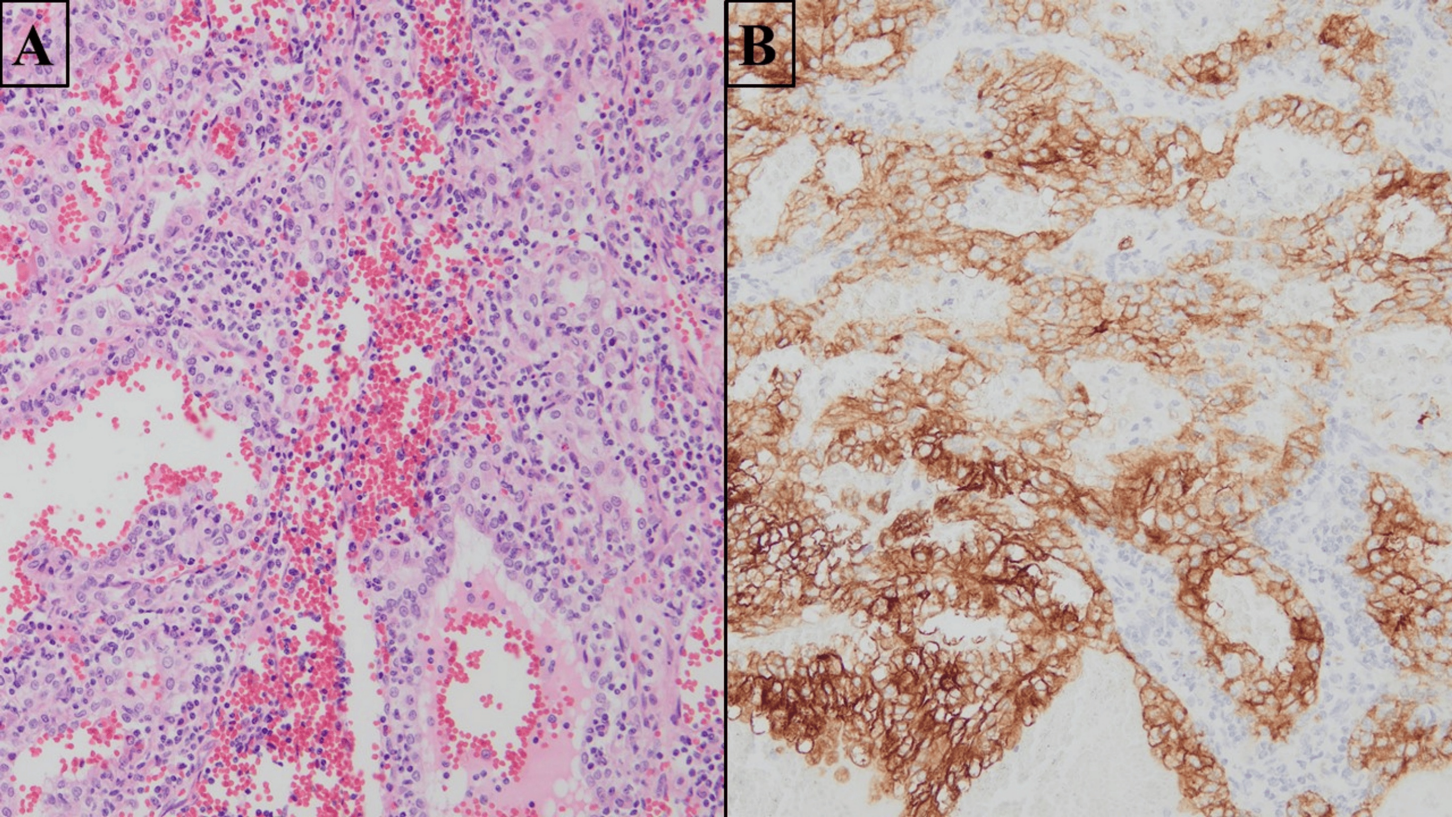
Patient's Initial ccRCC Tumor from Right Radical Nephrectomy A. H&E, 200x. The tumor shows clear cells with prominent nucleoli amidst a hemorrhagic background. B. IHC for CD10, 200x. The tumor cells show diffusely positive membranous staining for CD10, supporting the diagnosis of ccRCC. IHC: Immunohistochemistry; ccRCC: clear cell renal cell carcinoma

Gross examination demonstrated a well-defined, golden-yellow mass measuring 7.0 x 2.4 x 1.5 cm and confined within the spermatic cord and primarily within spermatic cord veins (Figures 2, 3). Microscopic examination of the specimen revealed a vascular tumor comprising cells with clear cytoplasm and delicate chicken-wire vasculature. Although the majority of the tumor was contained within the lumen of veins, there were focal areas of the tumor pushing and breaking through venous wall into the adjacent stroma (Figures 4A, 4B). Lymphocytic reaction within and around the tumor was also present. Histologic grade was predominantly grade two with focal areas of grade three (30%). Surgical margins were negative. This tumor morphology was similar to the patient’s known prior diagnosis of ccRCC, which showed positive staining for CD10 and PAX8 on radical nephrectomy (Figures 5A, 5B). The testis was uninvolved and showed focal atrophy with markedly reduced spermatogenesis. No other pathology in the specimen was present. The contralateral testis was unremarkable clinically and radiologically. No other metastases were detected, and the prior surgery bed was unremarkable.

The patient is currently being treated with adjuvant pembrolizumab immunotherapy and is doing well at three months follow-up. 

## Discussion

We present a rare case of unifocal ccRCC metastasis to the contralateral spermatic cord three years after initial diagnosis and surgery. From a clinical and radiological perspective, this patient's presentation had a differential which included varicocele, spermatocele, torsion, and neoplasm. The utilization of ultrasound was crucial in narrowing this differential, aiding in the clinical decision for radical orchiectomy due to suspicion of neoplasm. As previously discussed, RCC displays frequent metastases in approximately one-third of cases [1]. Despite the frequency of metastasis in RCC, contralateral and retrograde spermatic cord metastases of ccRCC are extremely rare, with only one prior reported case. In the literature, two prevalent theories for the route of retrograde metastasis have been proposed. 

The first of the theorized routes is the extension of the tumor through the left renal vein and into the spermatic cord vein. The left spermatic vein directly connects to the renal vein as opposed to the right spermatic vein, which drains into the IVC. Conceptually, this anatomy facilitates easier extension and metastasis of ccRCC from the left renal vein down into the left spermatic cord. There are case reports that support this theory. Mabjeesh et al. described a case of a left RCC renal vein thrombus extending directly into the left spermatic vein, while Sweigert et al. similarly presented a case of left RCC extending directly into the left gonadal vein into the ovary [7,8]. Including our case, only five of the 14 reported cases of spermatic cord ccRCC metastases occurred in the right spermatic cord, and of those, four were ipsilateral.

The second of the theorized routes is that tumor cells in ccRCC spread through the vertebral venous plexus, formerly known as Batson’s plexus. First described by Oscar V. Batson in 1940, the vertebral venous plexus is an extensive, bidirectional system of valveless veins that connect deep pelvic, thoracic, and vertebral plexuses [9]. The tumor is thought to spread contralaterally through the vertebral venous plexus, then to contralateral renal capsular veins, and finally to the spermatic cord vessels [10,11]. Thus, the vertebral venous plexus provides a route for metastatic spread that is separate from the lymphatic system. Its role in the retrograde venous spread of malignancy is now well-described [12].

In our patient’s case, his primary tumor originated in the right kidney and spread contralaterally and retrograde to the left spermatic cord. One other case in the literature displays a similar retrograde, contralateral metastases of ccRCC to the spermatic cord, though this case demonstrated a primary tumor in the left kidney with spread to the right spermatic cord [13]. The extreme rarity of contralateral and retrograde spermatic cord metastases when compared to ipsilateral retrograde spermatic cord metastases suggests that perhaps direct extension through the renal vein is the more common route of metastasis. However, the existence of Batson’s plexus provides a potential explanation for this contralateral spread where direct extension through the renal and spermatic vein is less likely. 

## Conclusions

Retrograde and contralateral metastases in renal cell carcinoma are rare. When such metastases occur, they are thought to be anatomically facilitated by the vertebral venous plexus or by direct extension into the renal and gonadal veins. In discussing this case, we hope to bring awareness to this venous route facilitating the contralateral metastatic dissemination of ccRCC. Understanding anatomical correlations to pathological findings can help pathologists create a broad but logical differential when encountering tumors with unusual sites or presentations.
